# Effect of Zr Content on the Microstructure and Corrosion Behavior of 1235D Aluminum Alloy

**DOI:** 10.3390/ma19173657

**Published:** 2026-08-28

**Authors:** Jiming Li, Yanan Liu, Shoukun Li, Ertuan Zhao

**Affiliations:** 1 Shandong Binzhou Tuopu Chemical Engineering Co., Ltd., Binzhou 256600, China; 15305433168@163.com; 2 Binzhou Research Center for Hazardous Chemical Safety Science, Binzhou 256600, China; 3 School of Mechanical Engineering, Shandong University of Technology, Zibo 255409, China

**Keywords:** Zr content, 1235D aluminum alloy, microstructure, corrosion behavior, corrosion mechanism

## Abstract

This study investigated 1235D aluminum alloys with varying Zr additions to elucidate how Zr content affects the alloy microstructure and corrosion resistance. The results showed that Al_3_Zr precipitates refined the grains by pinning grain boundaries. The 0.06% Zr alloy exhibited the finest grain structure, the lowest fraction of low-angle grain boundaries, the lowest geometrically necessary dislocation density, and the fewest microstructural defects susceptible to corrosion. Electrochemical and immersion corrosion tests indicated that the corrosion resistance followed the order 0.06% Zr > 0.12% Zr > 0.02% Zr. The 0.06% Zr alloy exhibited the highest polarization resistance and formed a dense, stable passive film that effectively hindered Cl^−^ penetration, thereby minimizing matrix dissolution and the formation of loose hydroxide corrosion products. An insufficient Zr content provided limited grain refinement, whereas excessive Zr promoted precipitate agglomeration; both conditions intensified localized corrosion. Considering both microstructural evolution and corrosion behavior, 0.06% was identified as the optimal Zr addition for this alloy system. These findings provide a theoretical basis for optimizing the Zr content and improving the corrosion resistance of 1235D aluminum alloys in industrial production.

## 1. Introduction

With the rapid development of emerging industries such as renewable energy storage, advanced electronics manufacturing, and lightweight equipment production, increasingly stringent requirements have been placed on the comprehensive service performance of high-purity wrought aluminum alloys. Owing to their excellent formability, electrical conductivity, and mechanical properties, high-purity wrought aluminum alloys have become preferred materials for high-end industrial applications [1,2,3,4,5]. Among them, 1235D aluminum alloy is a key material for lithium-ion battery current collectors and electronic electrode foils and is widely used in the renewable energy, electronics, and light manufacturing industries [6,7,8].

However, 1235D aluminum alloy is susceptible to severe corrosion-related failure under practical service conditions. Corrosive species such as Cl^−^ and moisture are commonly present in battery operating environments, humid outdoor atmospheres, and industrial salt-spray conditions, where they can readily induce localized corrosion, particularly pitting [9,10,11,12]. Chloride-induced pitting is the predominant corrosion mode of this alloy. Such corrosion damage directly compromises the surface integrity of electrode foils and current collectors, increases interfacial contact resistance, and reduces electrical conductivity. These effects not only decrease battery energy efficiency and shorten cycle life but may also cause localized overheating, short circuits, and other safety hazards in severe cases. Consequently, corrosion significantly limits the long-term, reliable application of 1235D aluminum alloy in advanced renewable energy systems [13,14,15].

Microalloying is one of the most efficient, economical, and practical approaches for improving the performance of aluminum alloys. The addition of trace amounts of transition-metal elements enables precise control over microstructural evolution during solidification, rolling, and heat treatment, thereby promoting grain refinement, impurity removal, and the dispersed precipitation of secondary phases while enhancing corrosion resistance. Zr is regarded as one of the most effective microalloying elements for aluminum alloys because of its low solubility and strong precipitation tendency. It can form finely dispersed Al_3_Zr precipitates that refine grains, purify grain boundaries, and effectively improve the corrosion resistance of aluminum alloys [16,17,18,19]. To date, most related studies have focused on 2xxx-, 5xxx- and 7xxx-series aluminum alloys [20,21,22,23,24].

A series of existing studies have verified that trace Zr microalloying can refine grains and boost the corrosion resistance of aluminum alloys [25,26,27]. Nevertheless, most available literature targets structural aluminum alloys, and only limited research preliminarily explores Zr addition in pure aluminum or battery-grade aluminum foils. Previous works predominantly emphasize microstructure evolution and mechanical properties, while systematic comparisons of corrosion performance across varied Zr content gradients are still insufficient. Furthermore, few reports distinguish the distinct corrosion deterioration mechanisms induced by insufficient Zr and excessive Zr from the perspective of passive film stability and chloride-induced pitting. Whether a moderate Zr addition can stabilize passive films and suppress Cl^−^ attack in high-purity 1235D alloy still lacks systematic experimental demonstration. Up to now, systematic research clarifying how Zr content modulates the microstructure, corrosion behavior and underlying corrosion mechanisms of high-purity 1235D aluminum alloy remains scarce. The ambiguous modification mechanisms of Zr further hinder the development of high-performance 1235D aluminum alloys. Due to the low equilibrium solid solubility of Zr in Al within the Al-Zr binary system, three Zr concentrations (0.02%, 0.06% and 0.12%) were selected to explore their influences on the microstructure and corrosion resistance of 1235D alloy. The concentrations correspond to low Zr addition, the vicinity of critical precipitation concentration, and excessive Zr addition, respectively. Such design covers the evolution from insufficient precipitates to dispersed precipitates and then coarsened, agglomerated precipitates, which allows us to establish the correlation among Zr content, microstructure and corrosion performance.

Accordingly, 1235D aluminum alloys with varying Zr contents were prepared in this study. Scanning electron microscopy (SEM), energy-dispersive spectroscopy (EDS), electron backscatter diffraction (EBSD), and X-ray diffraction (XRD) were employed to systematically evaluate the effects of Zr content on grain morphology, grain-boundary characteristics, secondary-phase precipitation, and microstructural homogeneity. Potentiodynamic polarization, electrochemical impedance spectroscopy, and static immersion corrosion tests were further conducted to investigate the corrosion rates, corrosion morphologies, and electrochemical responses of the alloys with different Zr additions. The findings are intended to address the current lack of systematic research on microalloying-induced corrosion resistance in high-purity 1235D aluminum alloy and to provide essential experimental evidence and theoretical guidance for composition optimization, process development, and engineering applications of highly corrosion-resistant, long-service-life 1235D aluminum alloy for lithium-ion battery current collectors.

## 2. Experimental Materials and Methods

1235D aluminum alloy ingots with varying Zr contents were cast using a vacuum induction melting furnace. The ingots were subsequently homogenized at 480 °C for 4 h and then air-cooled to room temperature. Their chemical compositions are listed in Table 1.

Electrochemical measurements were conducted using a conventional three-electrode system on an OrigaFlex multichannel electrochemical workstation (Origalys, Lyon, France). The 1235D aluminum alloy specimens with varying Zr contents served as the working electrodes, with an exposed area of 1 cm^2^. A platinum sheet and an Hg/HgO electrode were used as the counter and reference electrodes, respectively, and a 3.5 wt.% NaCl solution was used as the electrolyte. The open-circuit potential was monitored for 3600 s, and subsequent measurements were performed after the potential had stabilized. Potentiodynamic polarization curves were recorded over a potential range of −1.2 to 0.5 V at a scan rate of 1 mV/s. Electrochemical impedance spectroscopy (EIS) measurements were conducted over a frequency range of 10^−2^–10^5^ Hz using a sinusoidal potential perturbation with an amplitude of ±10 mV. The impedance data were fitted using ZSimpWin software (V3.50).

After grinding and polishing, the specimens were immersed in a 3.5 wt.% NaCl solution for 6, 12, 36, 72, and 120 h. Their corrosion rates in the 3.5 wt.% NaCl medium were determined using the immersion weight-loss method. Three replicate specimens were tested under each condition, and the average value was reported as the final result. After immersion, the corrosion morphologies and corrosion products were examined using an FEI Quanta 400 SEM (Thermo Fisher Scientific, Hillsboro, OR, USA), while the phase compositions of the corrosion products were analyzed using a Rigaku SmartLab XRD (Rigaku, Tokyo, Japan). The microstructural evolution, including grain orientation, of the 1235D aluminum alloys with varying Zr contents was characterized by EBSD using an Oxford Instruments Symmetry detector (Oxford Instruments, Abingdon, UK), with a step size of 0.225 μm.

## 3. Results and Discussion

### 3.1. Microstructural Characterization

**Figure 1:**
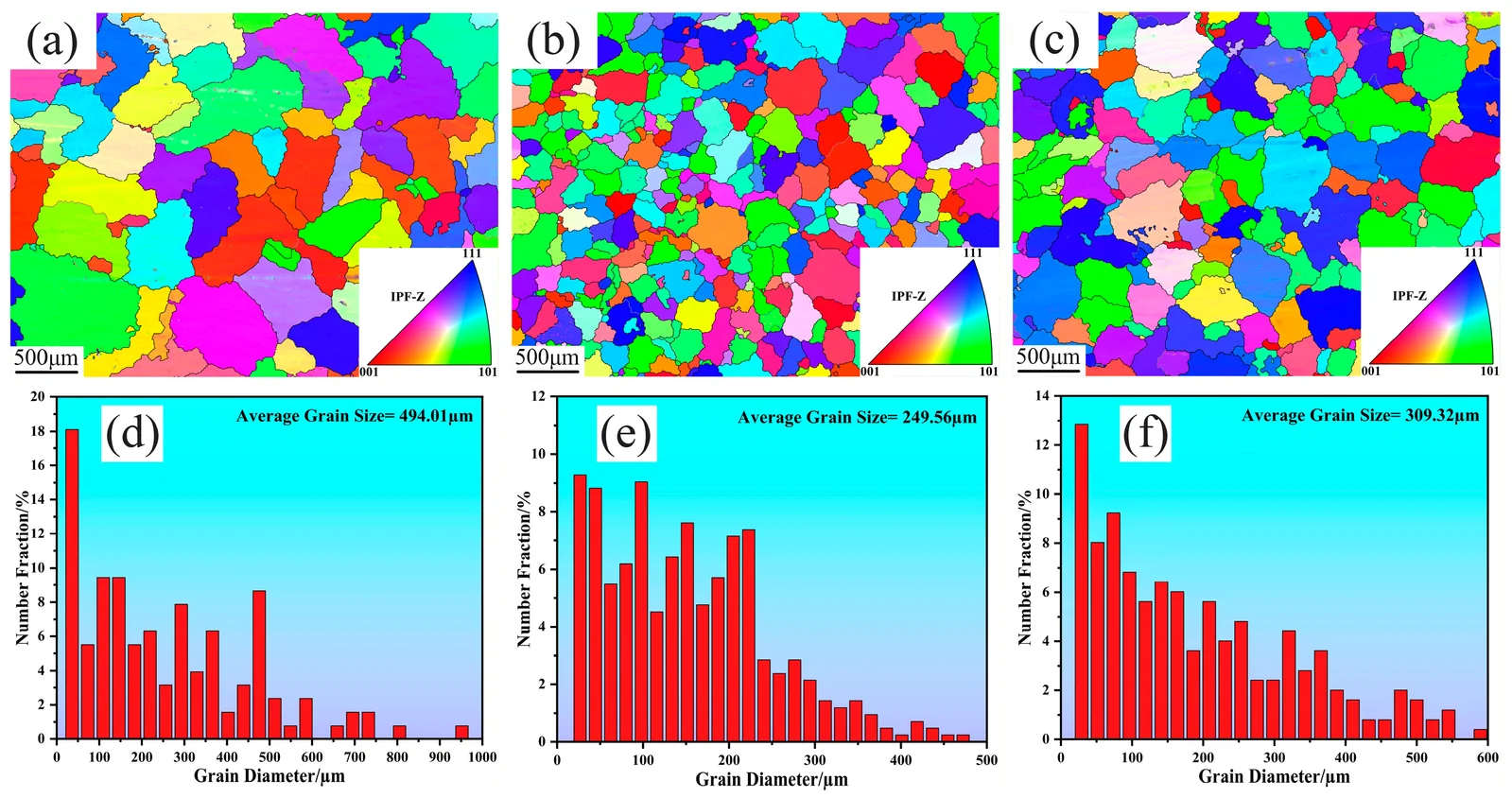
Inverse pole figure maps and average grain sizes of 1235D aluminum alloys (**a**,**d**) 0.02% Zr; (**b**,**e**) 0.06% Zr; (**c**,**f**) 0.12% Zr.

Figure 1 presents the microstructural characteristics of the 1235D aluminum alloys with varying Zr contents, as revealed by the EBSD inverse pole figure maps and grain-size distributions. As shown in Figure 1a,d, the 0.02% Zr alloy had an average grain size of 494.01 μm. The orientation map contained numerous coarse equiaxed grains larger than 600 μm, and the grain-size distribution was relatively broad, with a high proportion of large grains. As shown in Figure 1b,e, increasing the Zr content to 0.06% reduced the average grain size to 249.56 μm. The grains were finer and exhibited a narrower size distribution, with a higher proportion of small grains and no distinctly oversized grains. As shown in Figure 1c,f, when the Zr content was further increased to 0.12%, the average grain size increased to 309.32 μm. Nevertheless, severe grain coarsening remained suppressed, and the grain size was intermediate between those of the other two alloys.

**Figure 2:**
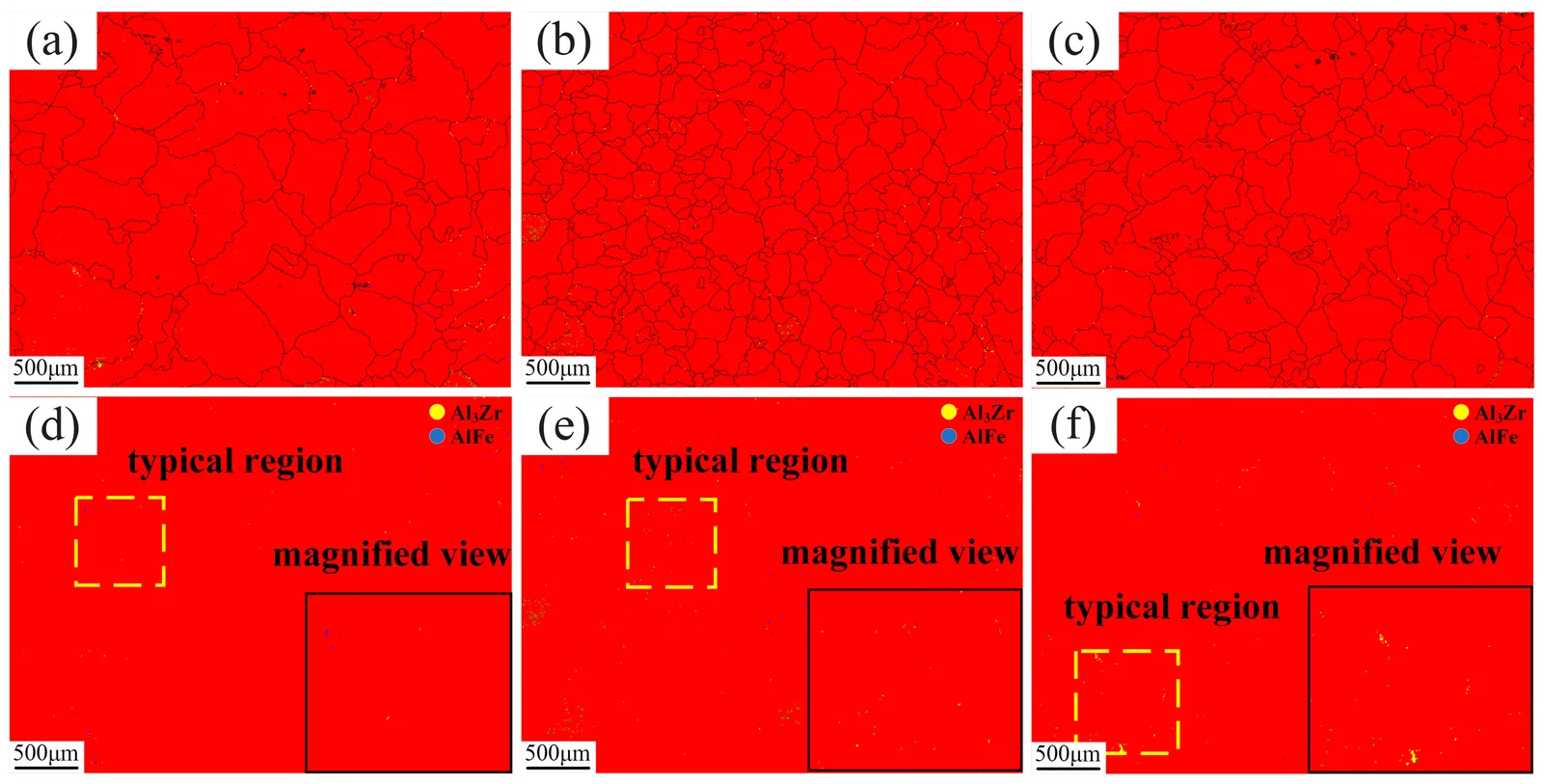
Phase diagrams of 1235D aluminum alloys (**a**,**d**) 0.02% Zr; (**b**,**e**) 0.06% Zr; (**c**,**f**) 0.12% Zr.

Figure 2 displays the phase distribution maps of 1235D aluminum alloys with various Zr contents. The red regions correspond to the Al matrix, the yellow markings denote Al_3_Zr precipitates, and the blue regions represent impurity phases such as AlFe. Typical regions highlighted by yellow dashed boxes in Figure 2d–f are magnified and presented within the black rectangular frames. At a Zr content of 0.02%, the Al_3_Zr precipitates were sparse and exhibited localized agglomeration and coarsening. Their low dispersion density resulted in a weak grain-boundary pinning effect, allowing rapid grain-boundary migration and grain coalescence during annealing. This ultimately promoted abnormal grain growth and increased the fraction of low-angle grain boundaries. At 0.06% Zr, fine Al_3_Zr particles were uniformly dispersed within the grains and along the grain boundaries, with no evident localized agglomeration. On the one hand, these finely dispersed precipitates exerted a sustained and strong grain-boundary pinning effect, effectively inhibiting grain-boundary migration and producing a uniform, fine-grained microstructure. On the other hand, the homogeneous distribution of fine secondary-phase particles dispersed potential galvanic corrosion sites and prevented localized corrosion from becoming concentrated. When the Zr content was increased to 0.12%, the greater supersaturation of Zr in the matrix promoted the precipitation of a high density of uniformly distributed Al_3_Zr dispersoids, thereby restoring a strong grain-boundary pinning effect. However, excessive Zr also promoted the coarsening and agglomeration of a small fraction of the Al_3_Zr particles. Consequently, the grain-refinement and microstructural-homogenization effects were somewhat weaker than those observed in the alloy containing 0.06% Zr.

**Figure 3:**
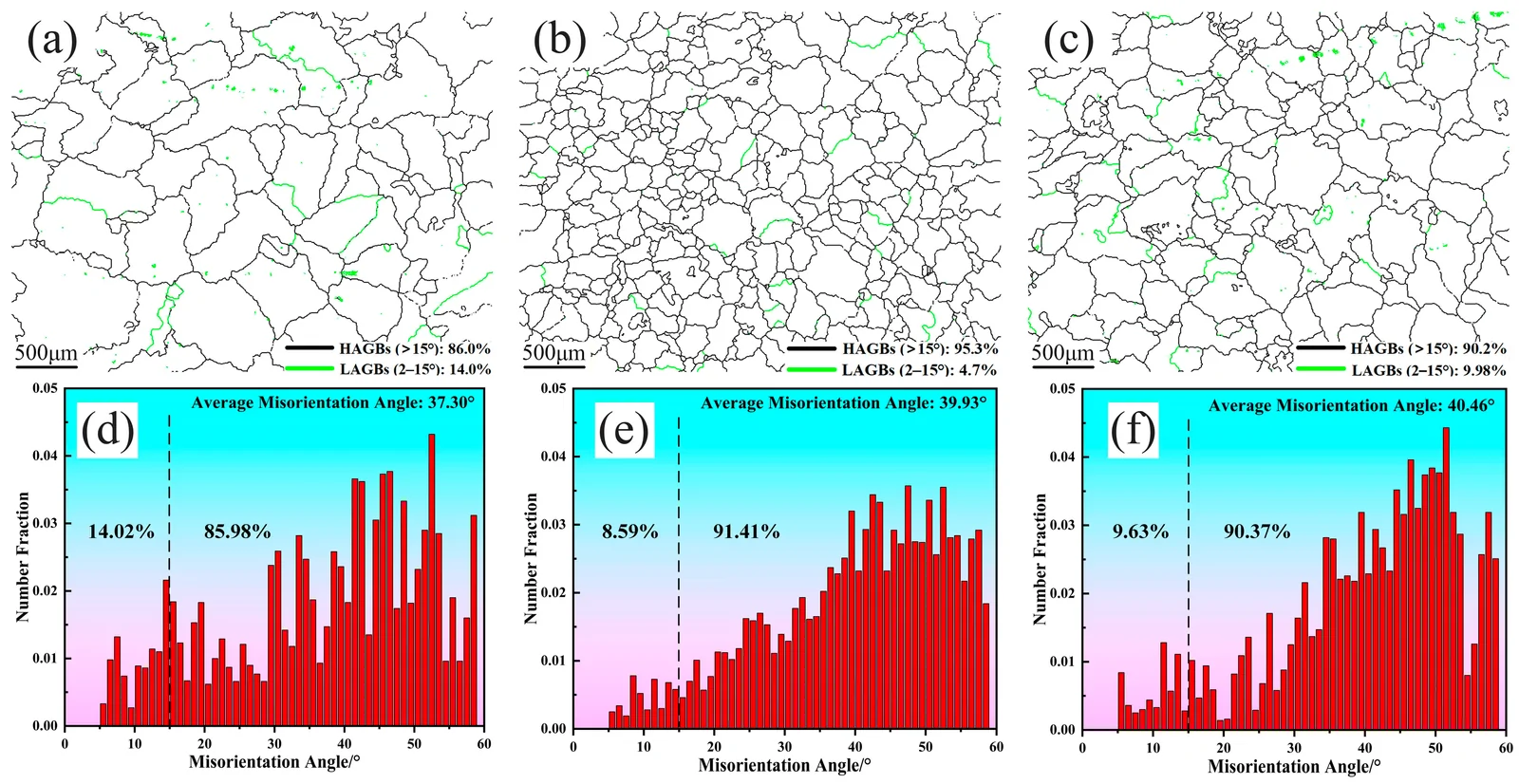
Grain boundary maps and misorientation angle distributions of 1235D aluminum alloys. (**a**,**d**) 0.02% Zr; (**b**,**e**) 0.06% Zr; (**c**,**f**) 0.12% Zr.

Figure 3 presents the grain-boundary distribution maps and grain-boundary misorientation statistics of the 1235D aluminum alloys with varying Zr contents. High-angle grain boundaries (HAGBs) were defined as boundaries with misorientation angles greater than 15°, whereas low-angle grain boundaries (LAGBs) were defined as those with misorientation angles between 2° and 15°. In Figure 3a–c, HAGBs and LAGBs are indicated by black and green lines, respectively. At 0.02% Zr, the fractions of LAGBs and HAGBs were 14.0% and 86.0%, respectively, and the average grain-boundary misorientation was 37.30°. Numerous fine, densely distributed low-angle subgrain boundaries were observed, indicating that the precipitates provided insufficient grain-boundary pinning at this Zr level. The large number of LAGBs contained high dislocation densities and exhibited substantially greater electrochemical activity than the matrix. Their adverse effect outweighed any potential benefit associated with the coarse-grained structure, making them preferential pathways for the penetration of corrosive species. At 0.06% Zr, the fractions of LAGBs and HAGBs were 4.7% and 95.3%, respectively, and the average grain-boundary misorientation increased to 39.93°. The microstructure was dominated by HAGBs. Uniformly dispersed, fine Al_3_Zr particles effectively regulated subgrain rotation and coalescence, promoting the transformation of most subgrain boundaries into relatively corrosion-resistant HAGBs and thereby reducing the driving force for localized corrosion initiation at the microscale. When the Zr content was increased to 0.12%, the fractions of LAGBs and HAGBs were 9.98% and 90.2%, respectively, and the average grain-boundary misorientation reached 40.46°. The high density of Al_3_Zr particles resulting from the increased Zr supersaturation strengthened grain-boundary pinning, promoted subgrain rotation and coalescence, and facilitated the transformation of subgrain boundaries into stable HAGBs.

To further investigate the evolution of grain boundaries in 1235D aluminum alloys with various Zr contents, the geometrically necessary dislocation density (ρ^GND^) at each pixel was calculated pixel-by-pixel using strain gradient theory based on EBSD-KAM (Kernel Average Misorientation) data. The step size adopted for EBSD mapping was 0.225 μm. Aluminum possesses an FCC crystal structure with a standard lattice constant a = 0.4047 nm. The stable full dislocation Burgers vector of FCC Al is a/2<110>. According to the vector magnitude formula, the magnitude of the Burgers vector in the Al matrix is calculated to be 0.286 nm. Based on strain-gradient theory, the GND density was calculated using the following equation [28,29]:

(1) $$\rho^{GND}=\frac{2\theta }{ub}$$

where *ρ^GND^* is the GND density, *θ* is the average local misorientation, *u* is the EBSD step size of 0.225 μm, and *b* is the magnitude of the Burgers vector.

**Figure 4:**
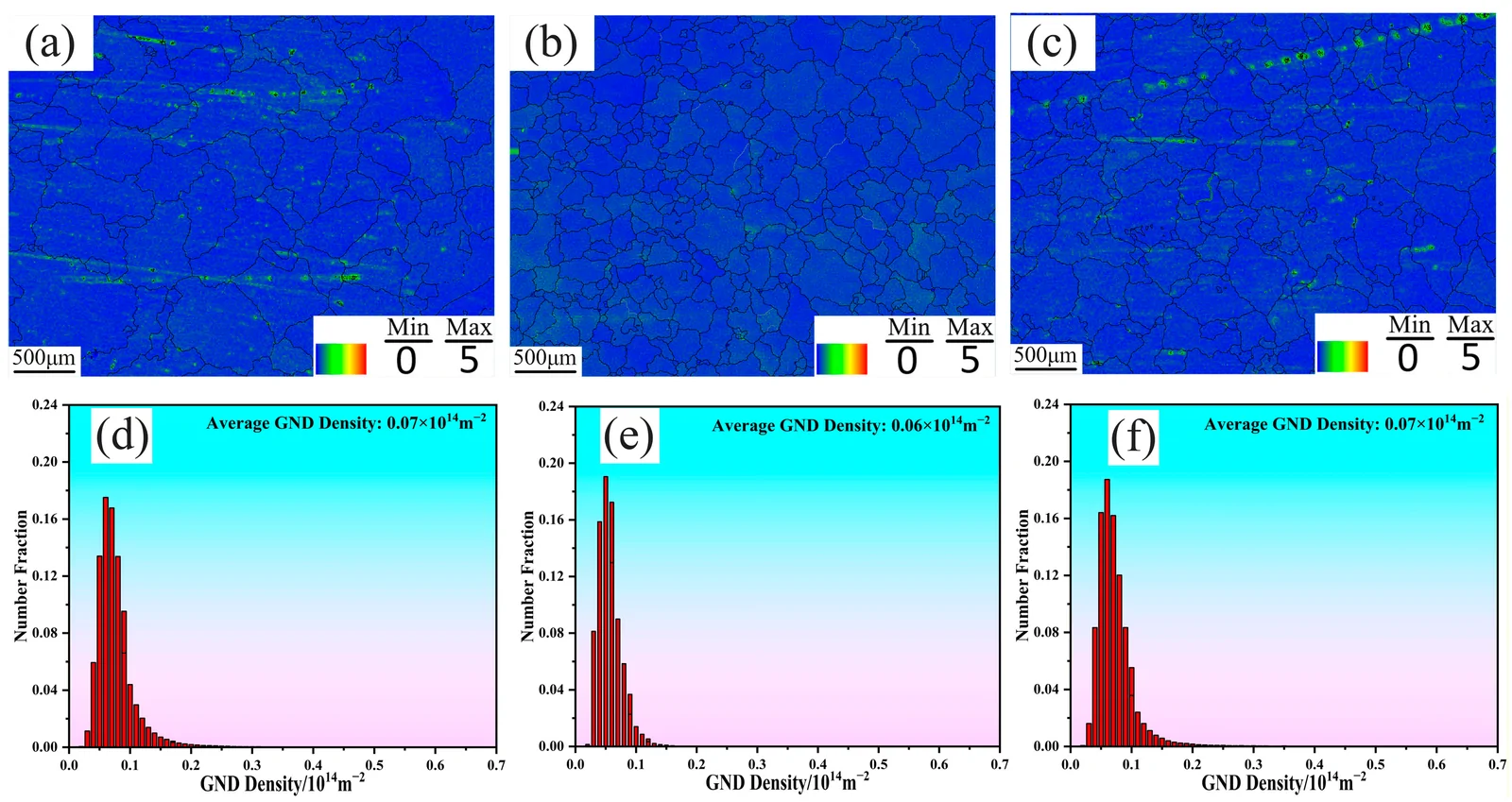
GND density maps of 1235D aluminum alloys (**a**,**d**) 0.02% Zr; (**b**,**e**) 0.06% Zr; (**c**,**f**) 0.12% Zr.

Figure 4a–c presents the GND contour maps, which illustrate the spatial distribution of instantaneous local GND density at each measured pixel for specimens with three different Zr contents. The blue background represents regions with low dislocation density, whereas the bright-colored regions correspond to areas of GND accumulation. Figure 4d–f presents statistical histograms, and the labeled values in the figures correspond to the arithmetic mean of GND density calculated from all valid pixels within the entire EBSD mapping area. As shown in Figure 4a, the 0.02% Zr specimen contains only a limited number of Al_3_Zr precipitates, some of which exhibit localized coarsening and agglomeration. Consequently, their ability to pin and constrain dislocations is significantly weakened, allowing dislocations to undergo extensive slip, rearrangement, and annihilation. As a result, the overall GND distribution is relatively uniform, although isolated dislocation accumulations remain near a small number of incompletely evolved subgrain boundaries. In the 0.06% Zr specimen, fine Al_3_Zr precipitates are uniformly and dispersively distributed throughout the matrix. These precipitates effectively regulate dislocation motion and suppress excessive localized dislocation accumulation. Meanwhile, their homogeneous distribution promotes more uniform strain accommodation and facilitates dislocation rearrangement and annihilation during recovery. Therefore, the fewest bright GND-enriched regions are observed in Figure 4b, indicating the lowest degree of localized dislocation pileup and a relatively homogeneous dislocation distribution. When the Zr content is further increased to 0.12%, a high density of Al_3_Zr particles precipitates in the matrix, reestablishing a strong pinning effect on dislocation motion. Some dislocations are therefore unable to continue slipping or annihilating and instead accumulate locally near grain boundaries and around the precipitates. Accordingly, scattered GND-enriched regions are observed in Figure 4c. Dislocations are highly electrochemically active sites in aluminum alloys. A higher local GND density generally corresponds to more severe dislocation pileup, greater lattice distortion, and higher stored energy, thereby increasing the susceptibility to localized pitting corrosion. Thus, the lower and more homogeneous GND density observed in the 0.06% Zr specimen helps reduce local electrochemical heterogeneity and contributes to improved resistance to pitting corrosion.

### 3.2. Electrochemical Corrosion

**Figure 5:**
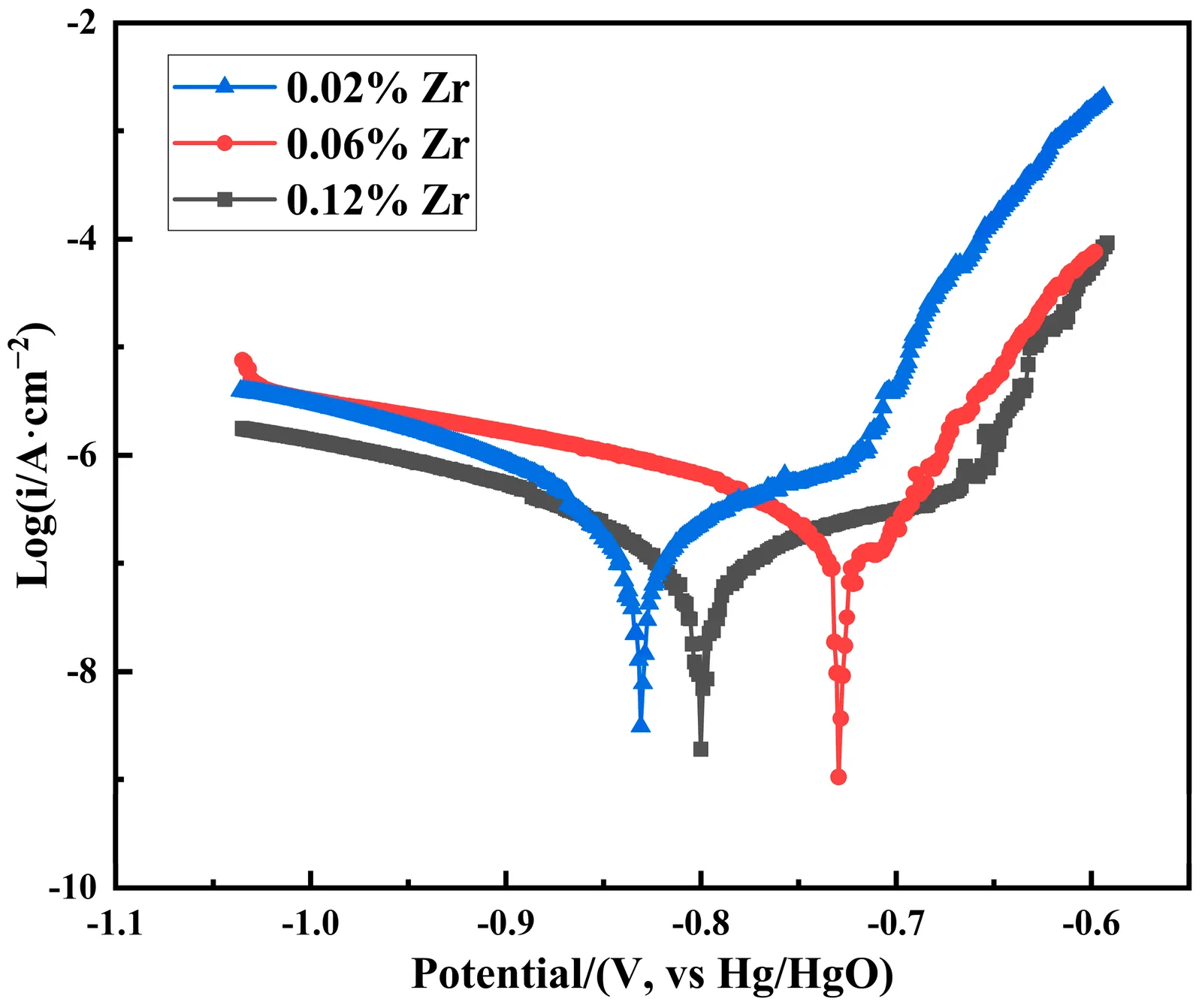
Potentiodynamic polarization curves of 1235D aluminum alloys with different Zr contents.

Figure 5 shows the potentiodynamic polarization curves of the 1235D aluminum alloys with different Zr contents. All three alloys exhibit typical active-dissolution polarization behavior. Among them, the 0.06% Zr alloy has the most positive corrosion potential and the lowest corrosion current density, followed by the 0.12% Zr alloy. In contrast, the 0.02% Zr alloy exhibits the most negative corrosion potential and the highest corrosion current density. Corrosion current density is a key parameter for evaluating corrosion rate. A lower corrosion current density indicates a slower anodic dissolution rate and, consequently, better corrosion resistance. An appropriate increase in Zr content can refine the grain structure, suppress the precipitation of impurity-containing phases, and reduce the driving force for interfacial microgalvanic corrosion in the aluminum alloy. However, when the Zr content is increased to 0.12%, the corrosion resistance decreases relative to that of the 0.06% Zr specimen. Excessive Zr addition may promote the formation of coarse Zr-containing intermetallic compounds, which act as new microgalvanic corrosion sites within the matrix and intensify localized corrosion.

Distinct passivation features can be observed in the anodic polarization region for the 0.02% Zr and 0.12% Zr specimens, whereas the 0.06% Zr sample fails to exhibit an evident passive plateau. This behavior originates from grain refinement induced by the appropriate Zr addition, which facilitates the formation of a denser and more stable passive film on the alloy surface and shifts the passive film breakdown potential toward the positive direction. Once the applied anodic potential reaches the breakdown potential, the passive film rapidly ruptures, pitting corrosion initiates quickly, and the current density rises continuously. Consequently, a wide and stable passive plateau cannot be detected on the polarization curve. In contrast, the passive films of the other two groups contain more defects and possess lower breakdown potentials. Local breakdown of passive films occurs in advance, leading to a coexistence state of partial passivation and localized pitting over a broad potential range, which yields a relatively flat quasi-passive region on the macroscopic polarization curves. This phenomenon demonstrates that the alloy with 0.06% Zr possesses a more stable passive film and stronger resistance to chloride-induced breakdown, rather than inferior corrosion resistance.

**Figure 6:**
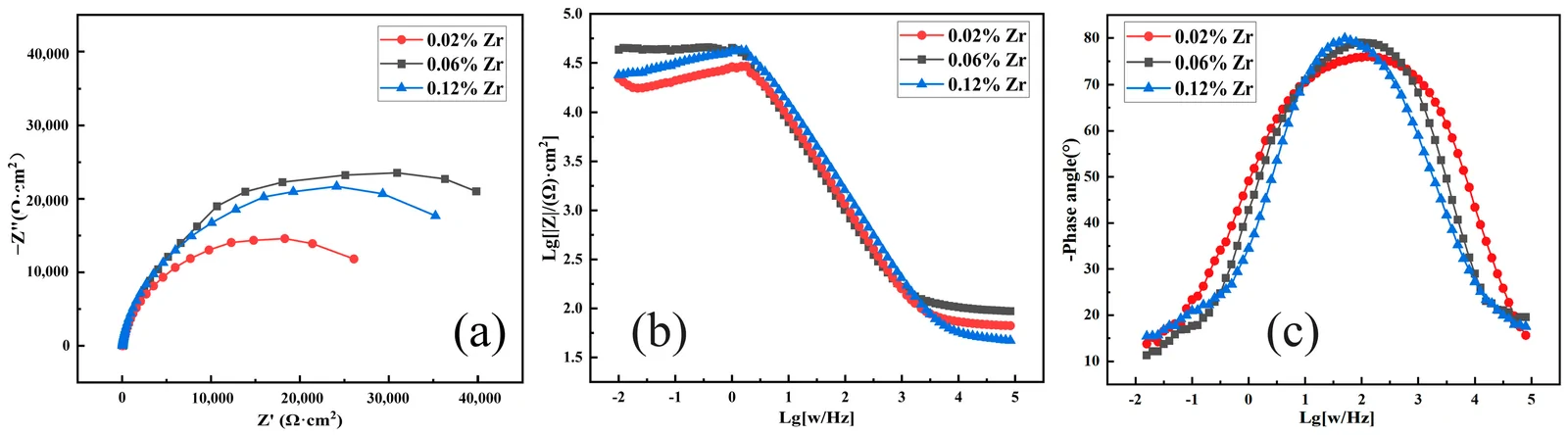
Electrochemical impedance spectra of 1235D aluminum alloys with varying Zr additions. (**a**) Nyquist plots; (**b**) Bode magnitude plots; (**c**) Bode phase angle plots.

Figure 6 presents the Nyquist plots, impedance-modulus Bode plots, and phase-angle Bode plots obtained from EIS measurements of the 1235D aluminum alloys with different Zr contents. In the Nyquist plots, all three curves exhibit a single, complete capacitive semicircle, indicating that the electrochemical systems are dominated by a single time constant associated with the interfacial reaction between the oxide passive film on the aluminum alloy surface and the corrosive medium. In general, the diameter of the capacitive loop provides a direct indication of the corrosion resistance of the surface passive film. A larger loop diameter corresponds to greater resistance to interfacial charge transfer and, therefore, better corrosion resistance. As shown in the figure, the capacitive-loop diameters follow the order 0.06% Zr > 0.12% Zr > 0.02% Zr, demonstrating that an appropriate Zr addition significantly improves the corrosion resistance of the alloy. As the Zr content increases, the loop diameter initially increases and then decreases, indicating that the corrosion resistance first improves and subsequently deteriorates.

**Figure 7:**
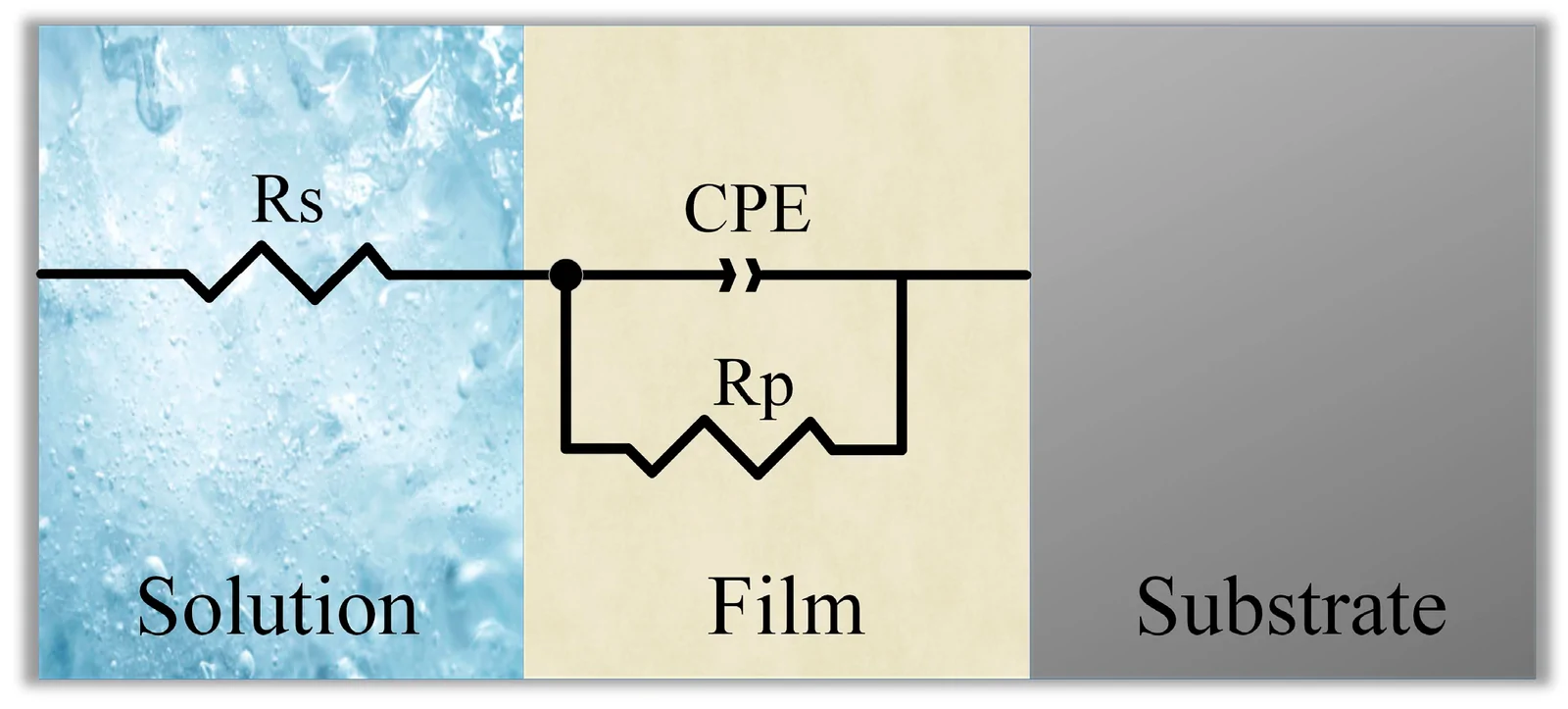
Schematic illustration of the equivalent circuit.

The Bode plots show relatively high values of log|Z| in the low-frequency region, while the maximum phase angles in the intermediate-frequency region approach 80° and remain high over a broad frequency range. These features indicate that relatively intact and stable alumina passive films formed on the surfaces of all three alloys. The equivalent circuit displayed in Figure 7 was adopted in this work to fit the EIS experimental data. R_s_ represents the solution resistance of the corrosive medium. The parallel branch consists of a CPE and polarization resistance R_p_, which characterizes the passive oxide film formed on the alloy surface. Real passive films are inherently inhomogeneous and contain pores and structural defects, deviating from ideal capacitive behavior. Accordingly, the CPE is used to replace an ideal capacitor to describe the capacitive response of the electrical double layer within the passive film. R_p_ reflects the capability of the passive film to hinder charge transfer and the diffusion of corrosive ions; a higher R_p_ value signifies superior barrier performance of the passive film. This equivalent circuit corresponds to a single time constant, which is consistent with the experimental observations of a single complete capacitive arc in the Nyquist plot and one distinct phase angle peak in the Bode plot. The quantitative parameters of each element in the equivalent-circuit diagram are listed in Table 2.

EIS fitting results show that the 0.06% Zr alloy exhibits the highest R_p_ value of 6.45 × 10^3^ Ω·cm^2^ among all three specimens. Moderate Zr addition produces prominent grain refinement and promotes a homogeneous, compact microstructure, which facilitates the development of a continuous, intact passive film and effectively reduces the area of exposed active substrate. In comparison, the 0.02% Zr alloy possesses coarse grains with limited grain boundary area and sparse precipitates, hindering the uniform growth of the passive film. For the 0.12% Zr alloy, excess Zr triggers the precipitation of coarse intermetallic particles. These particles introduce abundant microgalvanic interfaces within the matrix, disrupt the continuity of the passive film, and initiate localized microporous defects. Such defects accelerate charge transfer and chloride penetration, leading to a notable reduction in R_p_. The CPE is used to characterize the nonideal double-layer capacitive behavior of the alloy surface. Conversion and analysis of the CPE parameters using the Hsu-Mansfeld equation [30] indicate that a lower effective capacitance corresponds to a weaker capacitive response, a thicker passive film, and fewer surface defects. The 0.06% Zr specimen exhibits the lowest CPE-derived capacitance among the three specimens, indicating the greatest passive-film thickness and the lowest density of pores and defects. It therefore shows the best corrosion resistance. In contrast, the 0.02% Zr specimen has the highest CPE-derived capacitance, corresponding to a thinner passive film with a higher defect density and, consequently, the weakest protective capability.

### 3.3. Immersion Corrosion

**Figure 8:**
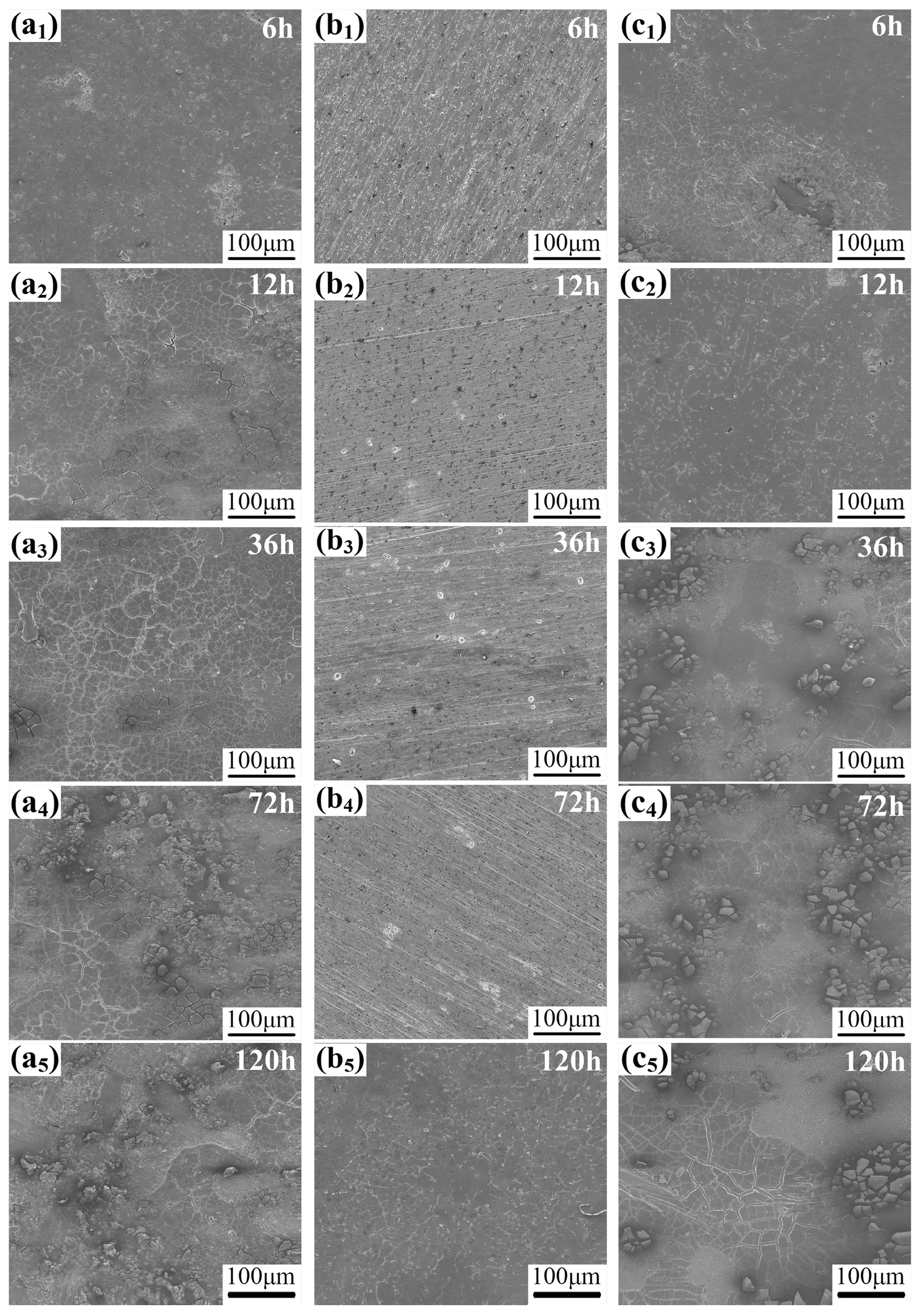
Corrosion morphologies of 1235D aluminum alloys with different Zr additions after immersion for various durations (**a_1_**–**a_5_**) 0.02% Zr; (**b_1_**–**b_5_**) 0.06% Zr; (**c_1_**–**c_5_**) 0.12% Zr.

Figure 8 shows the surface corrosion morphologies of 1235D aluminum alloys with different Zr contents after immersion in the corrosive medium for various periods. During the initial 6 h of immersion, shallow corrosion spots appeared locally on the 0.02% Zr specimen, whereas no obvious corrosion sites were observed on the surface of the 0.06% Zr specimen. In contrast, small corrosion pits formed locally on the 0.12% Zr specimen. After 12 h of immersion, a network of crack-like corrosion products developed on the surface of the 0.02% Zr specimen, and the corroded regions became extensively interconnected. The 0.06% Zr specimen exhibited only a small number of isolated, fine pitting sites, indicating a relatively slow corrosion propagation rate. For the 0.12% Zr specimen, the number of pits increased markedly, and localized corroded regions began to coalesce. After 36 h of immersion, the corrosion-product layer on the 0.02% Zr specimen became dense but severely cracked. Only a few additional pitting cavities formed on the 0.06% Zr specimen, while most of the substrate remained intact. By contrast, large amounts of agglomerated corrosion products accumulated in localized regions of the 0.12% Zr specimen, accompanied by severe pitting of the underlying substrate. During the prolonged corrosion stage at 72 and 120 h, extensive spallation of the corrosion-product film occurred on the 0.02% Zr specimen, with interconnected pores and cracks distributed across the surface, indicating widespread corrosion of the substrate. The 0.06% Zr specimen showed only slow growth of a limited number of fine pits, and no large-scale cracking or accumulation of corrosion products was observed even after 120 h. In comparison, through-thickness network cracks developed in the surface corrosion-product film of the 0.12% Zr specimen, resulting in extensive local exposure of the underlying substrate.

The tiny black spots observed in Figure 8(b_1_–b_3_) are not actively propagating pitting cavities. These features stem from Zr-based intermetallic precipitates inside the alloy. For the 0.06% Zr alloy, precipitates are fine and homogeneously dispersed. At the initial immersion stage, microgalvanic coupling arises between the Al matrix and precipitates, triggering slight interfacial dissolution. As a result, these precipitates appear as dark spots under SEM. After this transient micro-dissolution in the early stage, a continuous and intact passive film rapidly forms on the 0.06% Zr alloy, hindering further in-depth corrosion propagation into the matrix. Therefore, no continuous deep expansion of corrosion cavities is detected on the alloy surface after immersion for 72 h and 120 h. In this work, the term “pitting resistance” describes the ability of the alloy to restrain sustained in-depth corrosion and avoid the generation of destructive pitting craters. In contrast, the 0.02% Zr alloy gradually develops a network corrosion morphology, whereas substantial accumulation of corrosion products emerges on the 0.12% Zr alloy at later immersion stages, leading to more severe localized corrosion. It follows that the early-stage dark spots only correspond to weak, short-term interfacial reactions. The passive film can promptly suppress subsequent corrosion progression, preventing the evolution of detrimental pitting corrosion.

**Figure 9:**
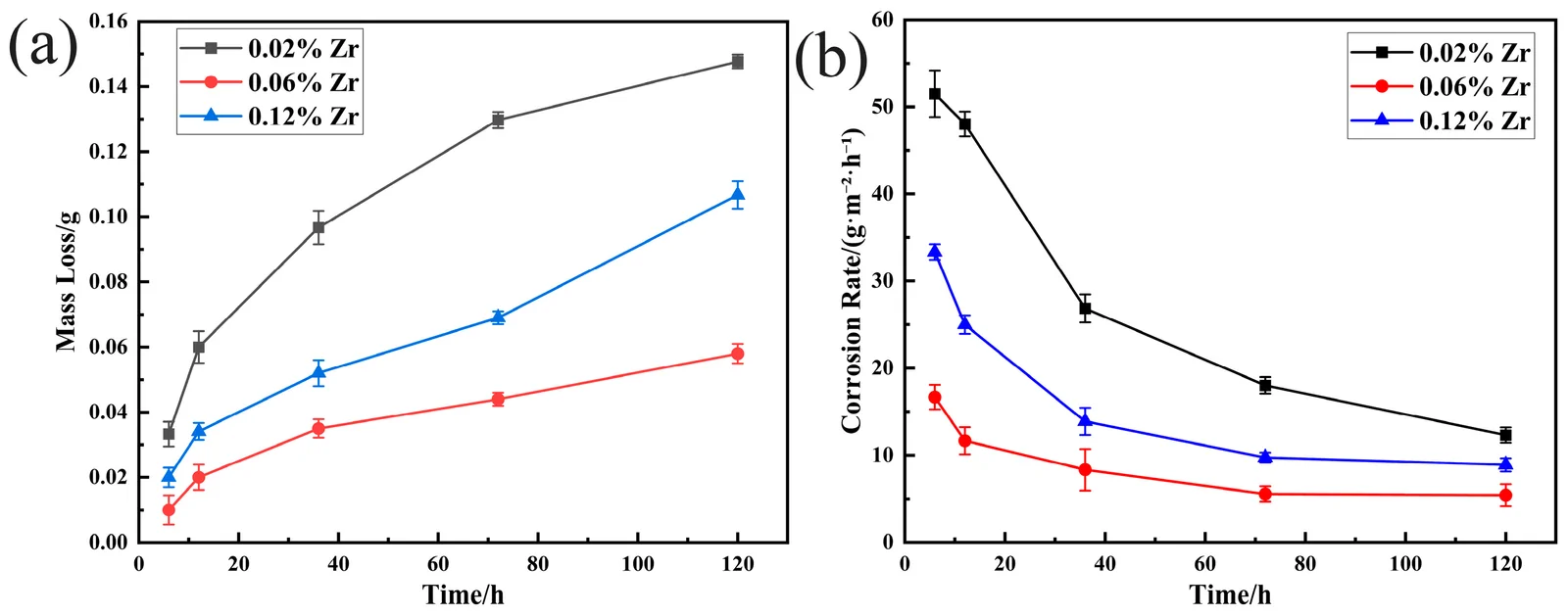
Mass loss and corrosion rate of 1235D aluminum alloys with varying Zr additions after immersion for different durations. (**a**) Mass loss; (**b**) corrosion rate.

Figure 9 presents the static immersion mass loss and corrosion rate curves of 1235D aluminum alloys with varying Zr contents after immersion for different durations. The mass loss data acquired from three parallel specimens at each immersion time are summarized in Table 3. As shown in Figure 9a, the mass loss of all three specimens increases continuously with immersion time. After 120 h of immersion, the total mass losses of the 0.02% Zr, 0.06% Zr, and 0.12% Zr specimens are 0.147, 0.058, and 0.108 g, respectively. The corrosion-rate curves in Figure 9b reflect the rate of metal dissolution per unit area and unit time. All three curves exhibit a continuous decrease with increasing immersion time. During the initial stage of immersion (0–40 h), a mixed corrosion-product film composed primarily of Al_2_O_3_ and aluminum salts rapidly forms on the specimen surfaces. This film partially isolates the substrate from the corrosive medium and suppresses the anodic dissolution reaction. During the later stage of immersion (60–120 h), the corrosion rates gradually stabilize as the corrosion-product films reach a dynamic equilibrium between formation and dissolution. Throughout the entire immersion period, the 0.06% Zr specimen consistently exhibits the lowest corrosion rate, with a steady-state value of only approximately 5 g·m^−2^·h^−1^ after 120 h. The corrosion rate of the 0.12% Zr specimen remains at an intermediate level. In contrast, the 0.02% Zr specimen shows the highest corrosion rate throughout the test, with an initial peak approaching 52 g·m^−2^·h^−1^ and a long-term steady-state rate of approximately 13 g·m^−2^·h^−1^.

**Figure 10:**
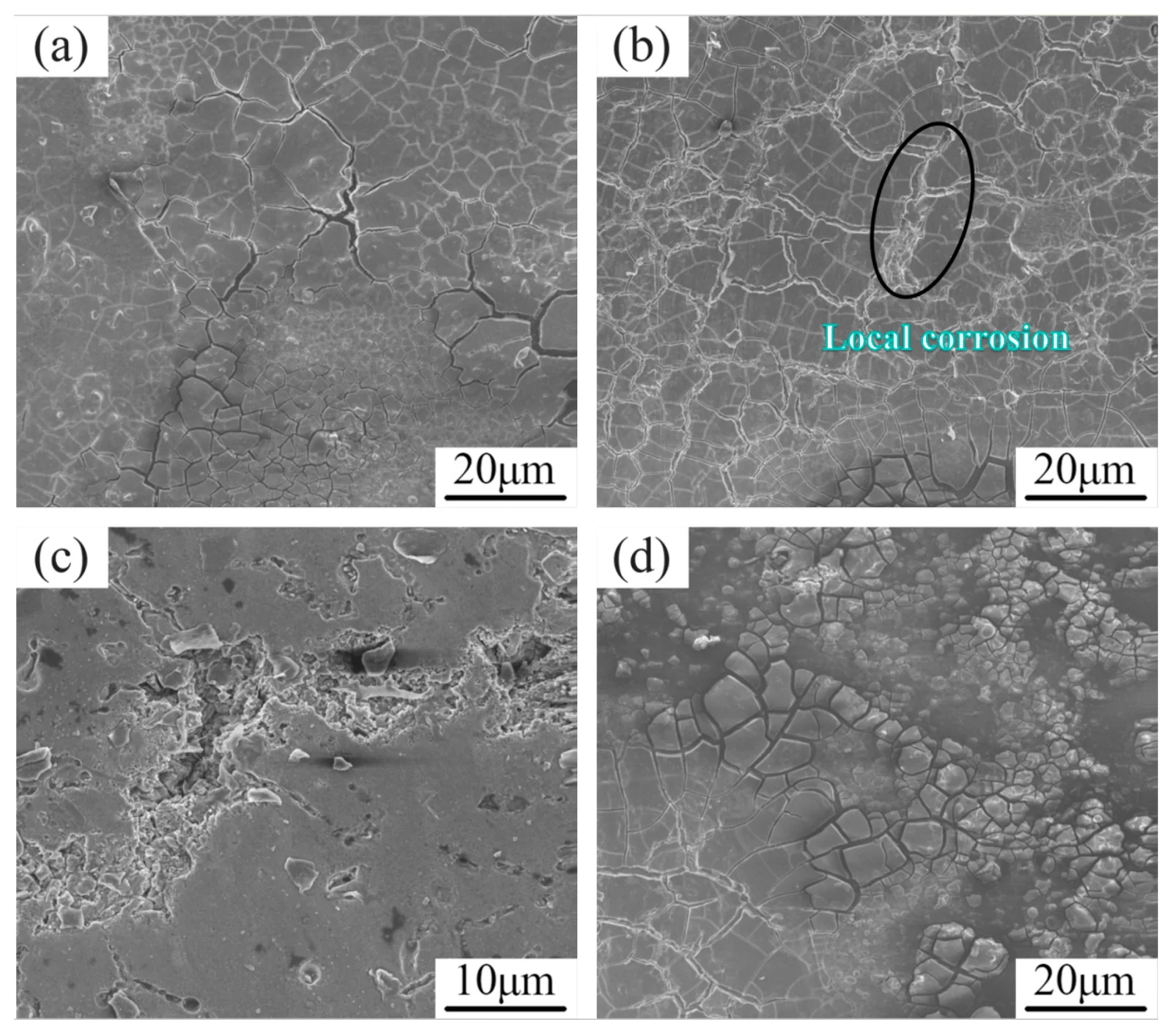
Corrosion evolution of 1235D aluminum alloy with 0.02 wt.% Zr during immersion. (**a**) Initial stage; (**b**) middle stage; (**c**) magnified local area; (**d**) later stage.

Figure 10 illustrates the corrosion evolution of 1235D aluminum alloy with 0.02% Zr. Figure 10a shows the surface morphology during the initial stage of corrosion. A continuous but relatively defective corrosion-product film forms on the specimen surface, with numerous fine, interconnected microcracks distributed throughout the film. Cl^−^ in the corrosive medium can penetrate through these microcracks and reach the underlying substrate. This penetration promotes the formation of occluded corrosion cells at the film-substrate interface, providing initial pathways for pit nucleation. Figure 10b shows the intermediate stage of corrosion, during which the preexisting network cracks continue to widen. Crack intersections and coalescence are clearly visible within the elliptical region. The corrosive medium continuously penetrates inward along these interconnected cracks, resulting in preferential dissolution of the local substrate and the formation of small subsurface pits beneath the corrosion-product film. The elliptically marked region in Figure 10b represents a typical active zone where passive film cracks intersect and localized corrosion preferentially initiates. Corrosive medium penetrates through these cracks in the passive film to the matrix interface, triggering the nucleation of localized corrosion. This site serves as the initiation position for pitting propagation. The magnified view of this region is displayed in Figure 10c, which enables clearer observation of corrosion pits and corrosion products. Figure 10c intuitively reveals loose flocculent corrosion products formed by matrix dissolution at the bottom of cracks. The original compact passive film has been completely fractured, exposing the metallic substrate directly to the corrosive medium and drastically accelerating the electrochemical reaction rate. Figure 10d shows the later stage of corrosion. The corrosion-product film undergoes extensive block-like cracking and spallation and breaks into large polygonal fragments. Substantial amounts of corrosion products accumulate in localized regions, while adjacent pits coalesce to form large, interconnected corroded areas. Consequently, the material undergoes sustained and progressive corrosion damage.

**Figure 11:**
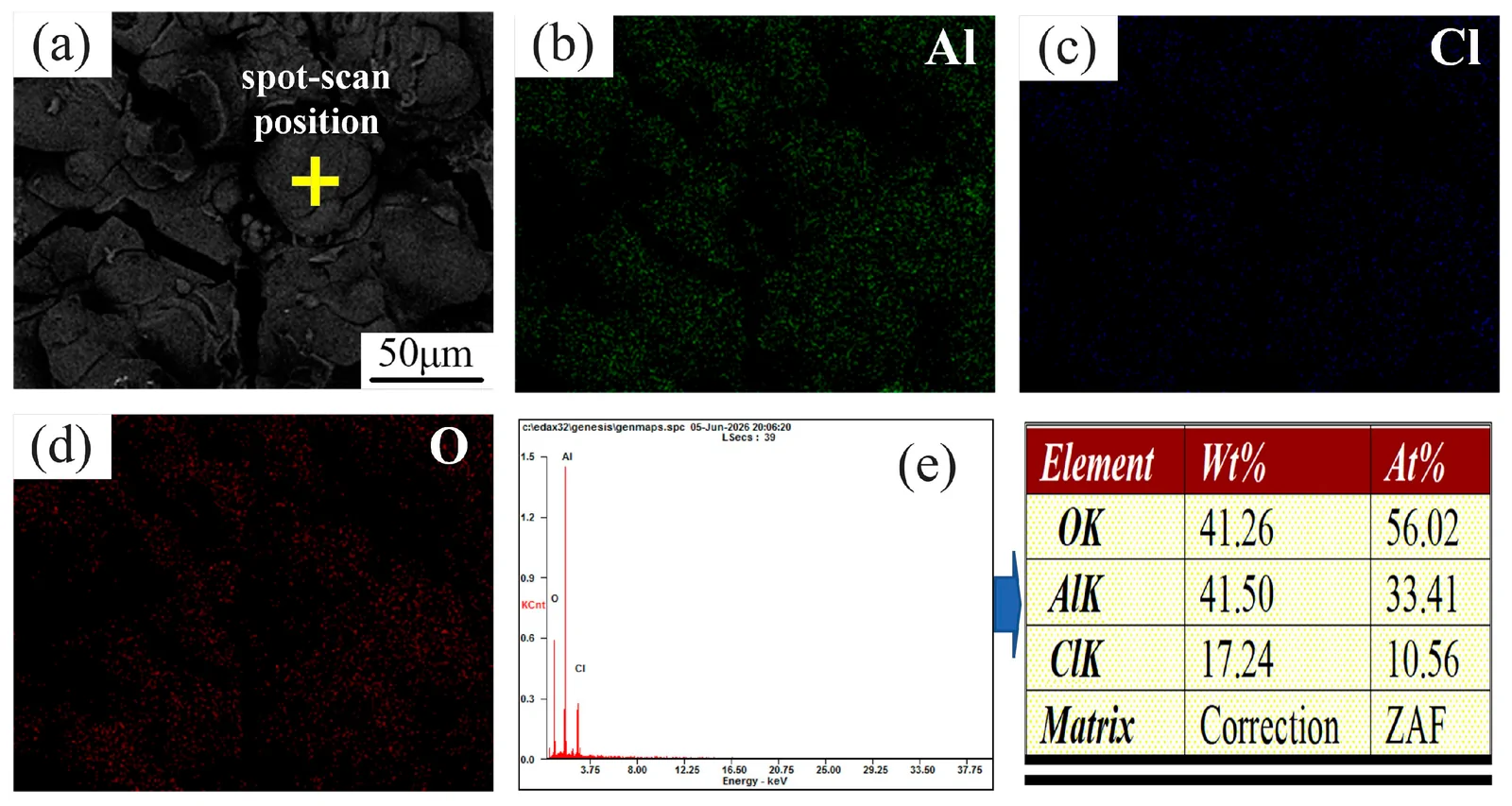
EDS elemental mapping of corrosion products on 1235D aluminum alloys. (**a**) Corrosion morphology; (**b**–**d**) surface scan images of Al, Cl, and O elements; (**e**) spot-scan results at the yellow plus-sign position in Figure (**a**).

Figure 11 shows the EDS elemental mapping and point-analysis results for corrosion products formed on the surface of 1235D aluminum alloy after 72 h of immersion in 3.5 wt.% NaCl solution. Figure 11a shows the SEM surface morphology of the alloy after corrosion. Numerous cracks are visible across the corrosion product layer, and the yellow cross denotes the location selected for EDS point analysis. Figure 11b–d display the elemental distribution maps of Al, Cl, and O, respectively. Aluminum is uniformly distributed, corresponding to the alloy substrate, while oxygen and chlorine tend to accumulate within the corrosion products and interconnected cracks. Figure 11e corresponds to the EDS point spectrum acquired at the yellow cross in Figure 11a, rather than an average spectrum obtained from area scanning over the entire field of view. The quantitative results reveal that the mass fraction of chlorine reaches 17.24% within the corrosion products. This confirms that chloride ions from the corrosive medium can penetrate the oxide film and become trapped within fissures of the film, establishing occluded corrosion cells that continuously degrade the substrate. After ingress, Cl^−^ reacts with the aluminum matrix to form chlorine-containing corrosion products. Volume expansion of these products further widens surface cracks, creating a self-accelerating cycle that exacerbates corrosion. In contrast, the denser passive film formed on the 0.06% Zr specimen effectively hinders chloride penetration and reduces chlorine accumulation at the metal-film interface.

**Figure 12:**
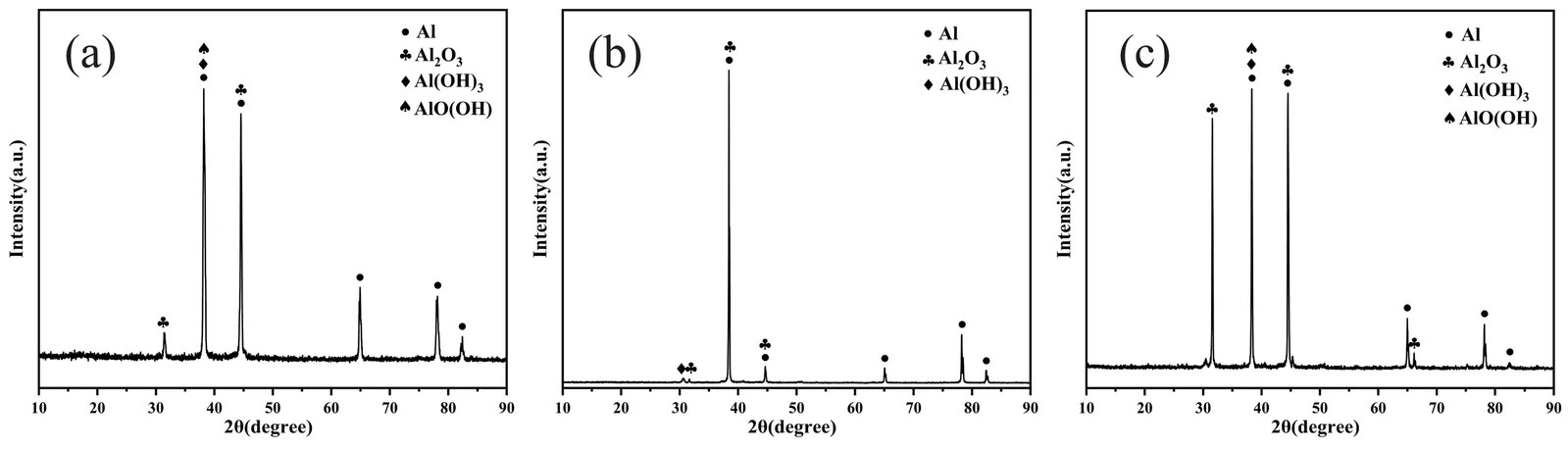
XRD patterns of corrosion products on 1235D aluminum alloys (**a**) 0.02% Zr; (**b**) 0.06% Zr; (**c**) 0.12% Zr.

Figure 12 shows the XRD patterns of corrosion products formed on aluminum alloys with three different Zr contents. Different symbols in the figure denote distinct corrosion product phases. The types of corrosion products are identical for all samples, consisting of the Al matrix, Al_2_O_3_, Al(OH)_3_ and AlO(OH), demonstrating that Zr addition does not alter the categories of corrosion products. For bulk specimens, XRD signals originate simultaneously from the surface corrosion product layer and the underlying substrate. Moreover, diffraction peaks of different phases overlap at certain 2θ positions, and the corrosion product layer exerts a shielding effect on signals from the substrate. Accordingly, direct comparison of absolute diffraction peak intensities is inappropriate. In this work, isolated characteristic peaks of the Al matrix free from peak overlap were selected as the reference. A semi-quantitative analysis was performed using the intensity ratio between characteristic peaks of hydroxide corrosion products and the Al matrix peaks. The results indicate that the 0.02% Zr alloy possesses the highest relative content of porous hydroxides, while the 0.06% Zr alloy exhibits the lowest fraction of hydroxides, and the value for the 0.12% Zr alloy lies between the two. Porous hydroxides provide weak protective capability. A higher relative content of such hydroxides implies more severe breakdown of the passive film and advanced development of localized corrosion.

In summary, uniformly dispersed Al_3_Zr precipitates govern alloy corrosion behavior via multiple mechanisms in addition to grain refinement. Firstly, Al_3_Zr precipitates possess a moderate potential offset relative to the Al matrix, tuning surface oxidation kinetics to promote the growth of a denser aluminum oxide passive film. This compact barrier restrains the adsorption and inward diffusion of aggressive Cl^−^ and postpones the onset of localized pitting corrosion. Secondly, microgalvanic coupling between the Al matrix and Al_3_Zr strongly relies on precipitate size, volume fraction and spatial distribution. While coarse intermetallics serve as cathodic sites to accelerate anodic dissolution of the adjacent matrix, nanoscale Al_3_Zr particles narrow the matrix-precipitate potential difference, homogenize interfacial current distribution and alleviate microgalvanic corrosion. Existing research confirms that dispersed nanosized Al_3_Zr enhances the pitting resistance of Al alloys [31]. However, excess Zr induces Al_3_Zr coarsening, which impairs passive film integrity and aggravates galvanic effects, degrading corrosion resistance [19,32]. Such discrepancies among published results stem from differences in alloy composition, fabrication processes and precipitate sizes, which also account for the corrosion performance trends of samples with varying Zr contents in this study.

## 4. Conclusions

This study investigated the effects of Zr content on the corrosion rate, corrosion morphology, and electrochemical corrosion behavior of 1235D aluminum alloy and clarified the mechanism by which Zr regulates the corrosion behavior of high-purity 1235D aluminum alloy. The main conclusions are as follows:

(1) Zr promotes the generation of dispersed Al_3_Zr precipitates. The 0.06% Zr alloy possesses the smallest grains. The specimen containing 0.02% Zr shows grain coarsening, whereas precipitate agglomeration occurs in the 0.12% Zr sample, deteriorating the grain refinement efficiency.
(2) The corrosion resistance followed the order 0.06% Zr > 0.12% Zr > 0.02% Zr. The 0.06% Zr alloy exhibited the highest polarization resistance and the lowest corrosion rate. Its dense surface passive film effectively inhibited the penetration of Cl^−^ ions.
(3) Subgrain boundaries, high dislocation densities, and agglomerated secondary phases increase the number of electrochemically active microgalvanic corrosion sites and accelerate pitting corrosion.
(4) 0.06% was identified as the optimal Zr addition for the 1235D aluminum alloy under the experimental conditions used in this study.

## Figures and Tables

**Table 1:** Chemical compositions of 1235D aluminum alloys with different Zr contents (wt./%).

Fe	Si	Cu	Ti	Zn	Sc	Zr	Al
0.40	0.10	0.10	0.02	0.01	0.02	0.02	Bal.
0.40	0.10	0.10	0.02	0.01	0.02	0.06	Bal.
0.40	0.10	0.10	0.02	0.01	0.02	0.12	Bal.

**Table 2:** Quantitative fitting parameters of the equivalent circuit model.

Sample	R_s_/Ω·cm^2^	R_p_/Ω·cm^2^	CPE/Ω^−1^·cm^−2^·s^n^	n_CPE_	Error/%
0.02% Zr	32.15	4.12 × 10^3^	9.12 × 10^−5^	0.788	4.549
0.06% Zr	40.23	6.45 × 10^3^	6.12 × 10^−5^	0.835	2.837
0.12% Zr	36.78	5.28 × 10^3^	8.56 × 10^−5^	0.802	4.113

**Table 3:** Mass loss data of parallel specimens after immersion for different durations.

Time/h	0.02% Zr	0.06% Zr	0.12% Zr
6	0.033 ± 0.002	0.010 ± 0.003	0.021 ± 0.002
12	0.060 ± 0.003	0.020 ± 0.002	0.034 ± 0.002
36	0.097 ± 0.004	0.034 ± 0.003	0.052 ± 0.003
72	0.130 ± 0.003	0.044 ± 0.002	0.069 ± 0.003
120	0.148 ± 0.002	0.058 ± 0.003	0.107 ± 0.004

## Data Availability

The original contributions presented in this study are included in the article. Further inquiries can be directed to the corresponding author.

## References

[1] Kang C., Ye K.F., Jia R.J., Zhou L. (2026). Construction of J-C intrinsic model based in high temperature dynamic mechanical response of 1235 aluminum plate. Integr. Ferroelectr..

[2] Yan W.D., Fu G.S., Chen H.L., Song L.L., Liu W. (2019). Texture characteristics of 1235 aluminum alloy after rolling. Mater. Technol..

[3] Zeng B., Chen C.K., Zhao H.L., Zhang C., Liu P., Wang G., He A., Liu F. (2025). A systematic review of the production of high-purity aluminum: Applications, preparation, and mechanisms. J. Mater. Res. Technol..

[4] Tan G.Y., Yue Y.C., Yang G., Zhou X., Ma N., He H.G., Yuan R. (2016). Evolution behavior of secondary phase particles in 1235 ultra-thin double zero foil blank during rolling process. Rare Met. Mater. Eng..

[5] Curtolo D.C., Xiong N., Friedrich S., Friedrich B. (2021). High- and ultra-high-purity aluminum, a review on technical production methodologies. Metals.

[6] Belit S., Sezen T., Basaran A., Ozel F., Lpek S.K. (2024). Thermomechanical and metallographic comparison of twin-roll CAST 1235, 3003, 8006, and 8079 alloy series used in the production of foil manufacturing. Light Metals.

[7] Karaaslan A. (2006). Influence of cold work and plate thickness on the gas-tungsten arc weldability of commercially pure aluminium. Mater. Test..

[8] Zhu Y.Z., Huang R.Y., Zhu Z., Xiang Z.D. (2011). Comparative study on effects of microstructures of hot rolled and twin roll casting 1235 aluminium alloy on surface quality of aluminium foils produced. Mater. Sci. Technol..

[9] Dong C.F., Xiao K., Xu L., Sheng H., An Y.H., Li X.G. (2011). Characterization of 7A04 Aluminum Alloy Corrosion under Atmosphere with Chloride Ions Using Electrochemical Techniques. Rare Met. Mater. Eng..

[10] Sun S.Q., Li C.L., Zheng Q.F., Wang X.M., Hu S.Q. (2017). The effect of environmental factors on long-term atmospheric corrosion behavior of commercially pure aluminum AA1035. Mater. Corros..

[11] Cui Z.Y., Ge F., Li X.G., Zhu M., Xiao K., Dong C.F., Wang X. (2017). Mechanistic studies of atmospheric corrosion behavior of Al and Al-based alloys in a tropical marine environment. J. Wuhan Univ. Technol.-Mater. Sci. Ed..

[12] Li T., Li X.G., Dong C.F., Cheng Y.F. (2010). Characterization of atmospheric corrosion of 2A12 aluminum alloy in tropical marine environment. J. Mater. Eng. Perform..

[13] Liu Y., Meng G.Z., Cheng Y.F. (2009). Electronic structure and pitting behavior of 3003 aluminum alloy passivated under various conditions. Electrochim. Acta.

[14] Yoshikawa D.S., Terada M., Assis S.L., Costa I., Phdilha A.F. (2016). Correlation between microstructure and corrosion behavior of two Al–Fe–Si alloys. Mater. Corros..

[15] Mazhar A.A., Arab S.T., Noor E.A. (2001). The role of chloride ions and pH in the corrosion and pitting of Al–Si alloys. J. Appl. Electrochem..

[16] Fadayomi O., Sanders P.G., Odegard G.M. (2019). Microstructure and properties of precipitation-hardened Zr and Zn-Zr based aluminum alloys. J. Alloys Compd..

[17] Dong P., Mo W.F., Ouyang Z.Q., Ling K., Luo B.H., Bai Z.H. (2023). Microstructural evolution and corrosion mechanism of micro-alloyed 2024 (Zr, Sc, Ag) aluminum alloys. Corros. Sci..

[18] Leng J.F., Ren B.H., Zhao J.W., Zhang Y.J. (2021). Effect Er and Zr microalloying on aluminum alloy 7075 microstructure formation during homogenization. Met. Sci. Heat Treat..

[19] Zhu Q.Y., Teng X.Y., Duan Z., He X., Zhuo G.R., Leng J.F. (2025). Effect of Sc and Zr additions on microstructure, mechanical properties and corrosion behavior of 7075 aluminum alloy. Met. Sci. Heat Treat..

[20] Kairy S.K., Rouxel B., Dumbre J., Lamb J., Langan T., Dorin T., Birbilis N. (2019). Simultaneous improvement in corrosion resistance and hardness of a model 2xxx series Al-Cu alloy with the microstructural variation caused by Sc and Zr additions. Corros. Sci..

[21] Wu X.L., Sun M., Hong L., Wen S., Wei W., Gao K., Rong L., Xiong X., Huang H., Nie Z. (2024). Er-containing microalloyed aluminum alloys: A review. J. Mater. Sci..

[22] Song C.R., Dong B.X., Zhang S.Y., Yang H.-Y., Liu L., Kang J., Meng J., Luo C.-J., Wang C.-G., Cao K. (2024). Recent progress of Al-Mg alloys: Forming and preparation process, microstructure manipulation and application. J. Mater. Res. Technol..

[23] Rometsch P., Fourmann J., Elgallad E., Chen X.-G. (2023). Use of Sc to improve the properties of AA5083 cast and rolled products. Light Metals.

[24] Zhang Z.H., Xiong B.Q., Liu S.F., Zhu B.H., Zuo Y.T. (2014). Changes of microstructure of different quench sensitivity 7000 aluminum alloy after end quenching. Rare Met..

[25] Zhao B., Luo Z.R., Yin N.A., Zhang Z.N., Zhang X.Z., Zhou C.S., Wang S., Fang Z.G., Zhou D.S., Wang T.L. (2024). Al_3_Zr nanophase-enabled anti-corrosion mechanisms in high-strength Al alloy by scalable micro-alloying design. J. Mater. Sci..

[26] So Y.S., Lim J.M., Chuang N.T., Kim J.G. (2023). Effect of homogenization and precipitation heat treatments on localized corrosion of Al-Mn-Zr Alloy with Fe impurity. Corrosion.

[27] Zhang Y., Ding L.P., Zou Q., Su Y., Jia Z.H., Zhen F.C., Zhuang L.Z., Atrens A. (2025). Effect of Al_3_(Sc, Zr) dispersoids on the mechanical and corrosion behavior of Al-Mg-Sc-Zr alloys. J. Alloys Compd..

[28] Gao H., Huang Y., Nix W.D., Hutchinson J.W. (1999). Mechanism-based strain gradient plasticity-I. Theory. J. Mech. Phys. Solids.

[29] Ma X.L., Huang C.X., Moering J., Ruppert M., Hoppel H.W., Goken M., Narayan J., Zhu Y.T. (2016). Mechanical properties of copper/bronze laminates: Role of interface. Acta Mater..

[30] Hsu C.H., Mansfeld F. (2001). Technical Note: Concerning the conversion of the constant phase element parameter Y into a Capacitance. Corrosion.

[31] Hui L., Han X.D., Wei H., Sun C.Z., Zhang X.L., Wang G.L., Wang X.Y., Qiao Y.P., Shcheretskyi O., Volodymyr S. (2025). Study on corrosion performance of friction stir processed AA6082-4 wt.% Al_3_Zr In situ composites. J. Mater. Eng. Perform..

[32] Kozlova N.A., Nokhrin A.V., Chuvildeev V.N., Shadrina Y.S., Bobrov A.A., Chegurov M.K. (2024). The Effect of a Sc:Zr ratio on the corrosion resistance of cast Al–Mg alloys. Phys. Met. Metallogr..

